# Enhancement of Nonlinear Dielectric Properties in BiFeO_3_–BaTiO_3_ Ceramics by Nb-Doping

**DOI:** 10.3390/ma15082872

**Published:** 2022-04-14

**Authors:** Ziqi Yang, Bing Wang, Yizhe Li, David A. Hall

**Affiliations:** Department of Materials, University of Manchester, Manchester M13 9PL, UK; ziqi.yang@postgrad.manchester.ac.uk (Z.Y.); bing.wang@manchester.ac.uk (B.W.); yizhe.li@manchester.ac.uk (Y.L.)

**Keywords:** bismuth ferrite–barium titanate, niobium doping, non-linear dielectrics, lead-free piezoelectric, ferroelectric, relaxor ferroelectric, core-shell microstructure, energy storage capacitors

## Abstract

BiFeO_3_–BaTiO_3_ (BF–BT) ceramics exhibit great potential for diverse applications in high temperature piezoelectric transducers, temperature-stable dielectrics and pulsed-power capacitors. Further optimization of functional properties for different types of applications can be achieved by modification of processing parameters or chemical composition. In the present work, the influence of pentavalent niobium substitution for trivalent ferric ions on the structure, microstructure and dielectric properties of 0.7BF–0.3BT ceramics was investigated systematically. Doping with niobium led to incremental reductions in grain size (from 7.0 to 1.3 µm) and suppression of long-range ferroelectric ordering. It was found that core-shell type microstructural features became more prominent as the Nb concentration increased, which were correlated with the formation of distinct peaks in the dielectric permittivity–temperature relationship, at ~470 and 600 °C, which were attributed to the BT-rich shell and BF-rich core regions, respectively. Nb-doping of BF–BT ceramics yielded reduced electronic conductivity and dielectric loss, improved electrical breakdown strength and enhanced dielectric energy storage characteristics. These effects are attributed to the charge compensation of pentavalent Nb donor defects by bismuth vacancies, which suppresses the formation of oxygen vacancies and the associated electron hole conduction mechanism. The relatively high recoverable energy density (W_rec_ = 2.01 J cm^−3^) and energy storage efficiency (η = 68%) of the 0.7BiFeO_3_–0.3BaTiO_3_ binary system were achieved at 75 °C under an electric field of 15 kV mm^−1^. This material demonstrates the greatest potential for applications in energy storage capacitors and temperature-stable dielectrics.

## 1. Introduction

In recent decades, research on energy storage materials has generated significant interest from academia and industry. Dielectric materials have been intensively investigated due to their fast charge/discharge rate, good performance under pulsed current/voltage conditions, excellent thermodynamic and mechanical stability and long cycling lifespan [1,2]. In general, dielectric materials can be divided into four categories; among these, relaxor ferroelectric (RF) and antiferroelectric (AFE) materials are the most suitable candidates for pulsed-power energy storage applications in view of their large maximum polarization, low remanent polarization and relatively low dielectric loss, in comparison with those of the linear dielectric and conventional ferroelectric categories [1,2,3]. Furthermore, the miniaturization and integration of passive components in electrical systems demands the development of novel dielectric materials with higher energy density, lower dielectric loss and excellent charge–discharge efficiency [1].

Generally, the total energy density (W_total_), recoverable energy density (W_rec_) and energy storage efficiency (η) are obtained by numerical integration of the polarization–electric field (P–E) loop according to the following formulae:

(1) $$W_{\mathrm{total}}=\int_{0}^{P_{m}}\mathrm{EdP}$$

(2) $$W_{\mathrm{rec}}=\int_{P_{r}}^{P_{m}}\mathrm{EdP}$$

(3) $$\eta =\frac{W_{\mathrm{rec}}}{W_{\mathrm{total}}}\times 100\%$$

where P_m_, P_r_ and E are the maximum polarization, remanent polarization and applied electric field, respectively. For future high-performance energy storage materials, the following three points need to be considered: high polarization difference (ΔP = P_m_ − P_r_), high breakdown strength (BDS) and sustainable, environmentally friendly compositions.

Recently, lead-free ceramics have attracted increasing attention to replace conventional lead-based dielectric materials, although the latter have been shown to exhibit excellent energy-storage properties. There are already numerous reports of lead-based dielectrics based on Pb(Zr, Ti)O_3_ (PZT), using various types of dopants and compositional adjustments to achieve antiferroelectric [4,5] or relaxor ferroelectric behavior [6,7]. For example, the W_rec_ value for (Pb, La)(Zr, Ti)O_3_ antiferroelectric ceramics was reported as 12.3 J cm^−3^ at an electric field of 42 kV mm^−1^ [4], while that for Mn-doped (Pb, La)(Zr, Sn, Ti)O_3_ (PLZST) relaxor thin films reached 31.2 J cm^−3^ at 200 kV mm^−1^ [6]. On the other hand, the total stored energy that can be achieved for a thin film device is restricted by its thickness and the number of dielectric layers that can be deposited reproducibly in combination with intermediate conductive electrodes.

Promising W_rec_ values have been reported in more environmentally-friendly, lead-free bulk ceramics, such as NaNbO_3_–(Bi_0.5_Na_0.5_)TiO_3_ (W_rec_~12 J cm^−3^ at 68 kV mm^−1^). Significant random fields are thought to occur in the relaxor AFE matrix in such NaNbO_3_-based compositions, which suppress the growth of nanodomains and the phase transition process, resulting in outstanding energy-storage properties [8]. In BiFeO_3_–BaTiO_3_–NaNbO_3_, the NaNbO_3_ substitution was found to improve the dielectric breakdown strength, resulting in W_rec_~8 J cm^−3^ at 36 kV mm^−1^ [9]. In (K_0.5_Na_0.5_)NbO_3_–SrTiO_3_ ceramics, the grain size was reduced into the submicron range by the addition of SrTiO_3_, leading to high BDS and high W_rec_ (W_rec_~4 J cm^−3^ at 40 kV mm^−1^) [10], while for Sm_0.03_Ag_0.91_NbO_3_ the Sm dopant was found to stabilize antiferroelectricity and increase the degree of disorder, resulting in slimmer P–E loops and improved recoverable energy density (W_rec_~5 J cm^−3^ at 29 kV mm^−1^) [11].

The various materials mentioned above represent some examples of the numerous studies on dielectrics for energy storage. Among the environmentally friendly lead-free materials, BiFeO_3_ (BF) was recognized as an important component due to its “giant” spontaneous polarization (>100 μm cm^−2^) [12,13]. By reducing the value of P_r_, BF-based compositions have the potential for a high polarization difference (ΔP), resulting in excellent energy storage performance. In particular, 0.7BF–0.3BT ceramics have been shown to exhibit high switchable polarization values (P_m_~44 μm cm^−2^ and P_r_~40 μm cm^−2^) due to the proximity of this composition to the morphotropic phase boundary (MPB) at 0.67BF–0.33BT [14]. However, the relatively low BDS of BF, which is generally associated with the volatilization of Bi_2_O_3_ and the changes in oxidation state of Fe^3+^ and Fe^2+^ ions during sintering, is another problem that needs to be addressed [15,16]. The BDS can be enhanced significantly by forming pseudo-binary or -ternary systems with perovskite compounds such as AgNbO_3_, SrTiO_3_, La(Mg_0.5_Ti_0.5_)O_3_ and Ba(Mg_1/3_Nb_2/3_)O_3_ [17,18,19,20,21].

It was found that Nb^5+^ substitution for Fe^3+^ on the B-site can dramatically increase the electrical resistivity of BiFeO_3_-based ceramics [22]. Moreover, the use of Nb as a donor dopant can promote the temperature stability of dielectric properties in the BF–BT solid solution with relaxor ferroelectric behavior [23]. The structural, dielectric, ferroelectric, magnetic and multiferroic properties in Nb doped BiFeO_3_-based ceramics were systematically investigated in recent years [24,25,26,27]. Some ternary systems were reported with high W_rec_, such as BiFeO_3_–BaTiO_3_–NaNbO_3_ [9] and BiFeO_3_–BaTiO_3_–Bi_0.5_Na_0.5_TiO_3_ [23]. However, the dielectric energy storage properties of the BF–BT binary system was given less attention, with one paper reporting that 0.65BiFeO_3_–0.35BaTiO_3_ with 3 mol% Nb_2_O_5_ addition yielded an improved W_rec_ of 0.71 J cm^−3^ at 9 kV mm^−1^ [28]. The potential of the BF–BT binary system is worthy of further exploration to obtain higher BDS and W_rec_ by modification of stoichiometry, selection of appropriate minor dopants and microstructural control.

In this work, a sequence of lead-free Nb-doped BF–BT ceramics were synthesized by solid state reaction and conventional sintering. A relatively high W_rec_ (2.01 J cm^−3^) and energy storage efficiency η (68.1%) were achieved for 0.7BiFe_0.98_Nb_0.02_O_3_–0.3BaTiO_3_ ceramics at an electric field level of 15 kV/mm and temperature of 75 °C. In addition, both W_rec_ and η are significantly improved at elevated temperatures, which indicates that Nb-doped BF–BT ceramics have the potential to be applied as energy storage dielectrics at elevated temperatures without property deterioration. Donor doping with niobium leads to a significant reduction in electronic conductivity, improvement of electrical breakdown strength and enhanced dielectric energy storage properties for BF–BT ceramics. 

## 2. Materials and Methods

### 2.1. Sample Preparation

0.7BiFe_1_–_x_Nb_x_O_3_–0.3BaTiO_3_ (x = 0, 0.005, 0.01, 0.02, 0.03, 0.04 and 0.05) ceramics were synthesized by the solid-state reaction method using Bi_2_O_3_ (99%), Fe_2_O_3_ (99%), BaCO_3_ (99%), TiO_2_ (99%) and Nb_2_O_5_ (99.99%) powders (Sigma-Aldrich, Gillingham, UK). The precursor powders were weighed out according to the required stoichiometry, followed by mixing and milling in propan-2-ol with yttria-stabilized zirconia media for 24 h and drying for 12 h at 90 °C. Afterwards, the powders were calcined at 800 °C for 4 h in air and milled again for 24 h. An addition of 2 wt% polyethylene glycol (PEG) lubricant was made into the dry calcined powders. Green pellets, 10 mm in diameter, were prepared by uniaxial pressing at a pressure of 100 MPa. The ceramic pellets were then sintered in a covered alumina crucible at 1010 °C for 3 h with heating and cooling rates of 5 °C/min. Conductive electrodes were applied using a silver paste (C2130823D1, Gwent Electronic Materials Ltd., Pontypool, UK), which was heated to 550 °C for 30 min for densification.

### 2.2. Microstructure Characterization and X-ray Diffraction

The sintered ceramic pellets were ground using 800, 1200, 2400 and 4000 grit SiC paper followed by polishing procedures with 3, 1, 0.25 μm diamond paste and 0.04 μm OPS silica colloidal suspension. Examination of microstructure and micro-chemical elemental mapping of the polished cross-sections were conducted using a scanning electron microscope (SEM, Tescan Mira3 SC, TESCAN-UK Ltd., Cambridge, UK) equipped with an energy-dispersive X-ray spectroscopy (EDS) system. The average grain sizes were determined quantitatively from the SEM images by line intercept analysis using ImageJ software (National Institutes of Health, Bethesda, MD, USA). X-ray diffraction (XRD) patterns of as-sintered samples were obtained using a PANalytical X’Pert Pro X-ray diffractometer (Malvern Panalytical Ltd., Malvern, UK) in the 2θ range from 10 to 100°, with a step size of 0.0167° and a counting time of 1.5 s for each step. TOPAS v5 software (Bruker Corporation, Billerica, MA, USA) was utilized for Rietveld refinement of the XRD data.

### 2.3. Electrical Property Measurements

Temperature-dependent dielectric measurements were conducted using an Agilent 4284A impedance analyzer (Agilent Technologies UK Ltd., Cheadle, UK) and a Carbolite CWF1200 horizontal tube furnace (Carbolite Gero Ltd., Hope, UK), using pure silver conductor wires for electrical contacts to the test specimens in the furnace. The dielectric constant and loss were determined at fixed frequencies of 1 kHz, 10 kHz and 100 kHz over the temperature range from 25 to 650 °C, using a single parallel capacitor–resistor network as the equivalent circuit element. Ferroelectric hysteresis measurements were conducted using a function generator (Agilent 33120 A, Agilent Technologies UK Ltd., Cheadle, UK) together with a Chevin Research HV amplifier. Four cycles of a sinusoidal electric field with a frequency of 2 Hz were applied to the samples. Polarization–electric field (P–E) loops were constructed from the recorded electric potential and induced current waveforms, according to the methods described by Stewart et al. [29]. W_total_, W_rec_ and η were obtained by numerical integration of the P–E loop, according to Equations (1) to (3) above. Five specimens of each composition were tested to provide statistically averaged polarization values and energy storage parameters. Typical uncertainties in the polarization, energy storage density and efficiency values were estimated as 0.01 C m^−2^, 0.01 J cm^−3^ and 0.05%, respectively.

## 3. Results and Analysis

### 3.1. Structure and Microstructure

**Figure 1:**
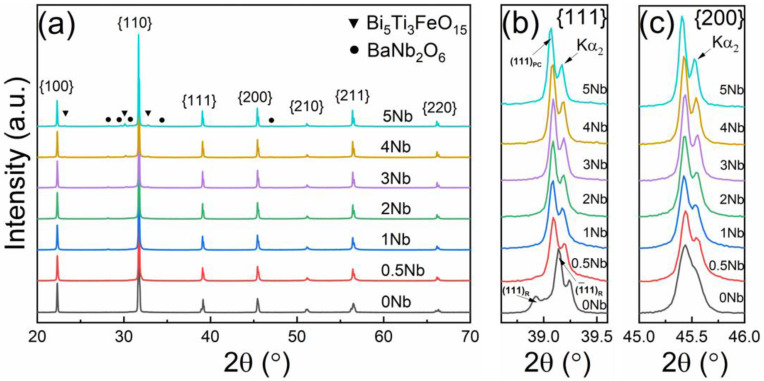
(**a**) Room temperature full X-ray diffraction patterns, (**b**) {111} peak profiles and (**c**) {200} peak profiles for the 0.7BF–0.3BT–xNb ceramics (0 to 5 at% Nb). Arrow labels in (**b**,**c**) indicate peaks associated with Cu Kα_2_ X-rays.

Figure 1 illustrates the XRD patterns of 0.7BF–0.3BT–xNb ceramics, measured at room temperature. Although a single rhombohedral phase was observed in the undoped BF–BT sample, doping with Nb induced a pseudo-cubic structure even at the lowest Nb dopant level of 0.5 at%. Additional diffraction peaks corresponding to Bi_5_Ti_3_FeO_15_ (ICSD_PDF#01-089-8545) [30] and BaNb_2_O_6_ (ICSD_PDF#01-077-0541) [30] were identified for Nb substitution levels of 1 at% and above, as shown Figure 1a. BaNb_2_O_6_ appears first, followed by Bi_5_Ti_3_FeO_15_, which may help to rebalance the stoichiometry of the perovskite solid solution. The {111} and {200} peak profiles, presented in Figure 1b,c, show slight shifts towards lower 2θ values with further Nb substitution, indicating a gradual increase in lattice parameters and unit cell volume.

The lattice parameters obtained by Rietveld refinement, summarized in Table 1, are also consistent with the direct observations of peak shifts in the XRD profiles. It has been suggested in previous work that the valence state of Nb could potentially change from Nb^5+^ (IR = 0.64 Å) to Nb^4+^ (IR = 0.68 Å) during sintering [31], resulting in lattice expansion due to the substitution of Nb^4+^ for Fe^3+^ (IR = 0.645 Å). Alternatively, the observed expansion of the lattice could possibly be caused by partial reduction of Fe^3+^ to Fe^2+^ ions, with an associated increase in ionic radius for Fe^2+^ (IR = 0.78 Å). The latter reaction can be represented by the following defect chemical equation, which involves charge compensation by the reduction of ferric to ferrous oxide in response to donor-type substitution of Nb^5+^ for Fe^3+^ in BiFeO_3_.

(4) $$3{\mathrm{Bi}}_{2}O_{3}+{\mathrm{Nb}}_{2}O_{5}+2{\mathrm{Fe}}_{2}O_{3}\to 6{\mathrm{Bi}}_{\mathrm{Bi}}^{x}+4{\mathrm{Fe}}_{\mathrm{Fe}}^{/}+2{\mathrm{Nb}}_{\mathrm{Fe}}^{••}+18O_{O}^{x}+O_{2}\left(g\right)$$

Further investigations are necessary to investigate the potential changes in the valence states of Fe^3+^ and/or Nb^5+^ ions in Nb-doped BF–BT ceramics during sintering.

**Figure 2:**
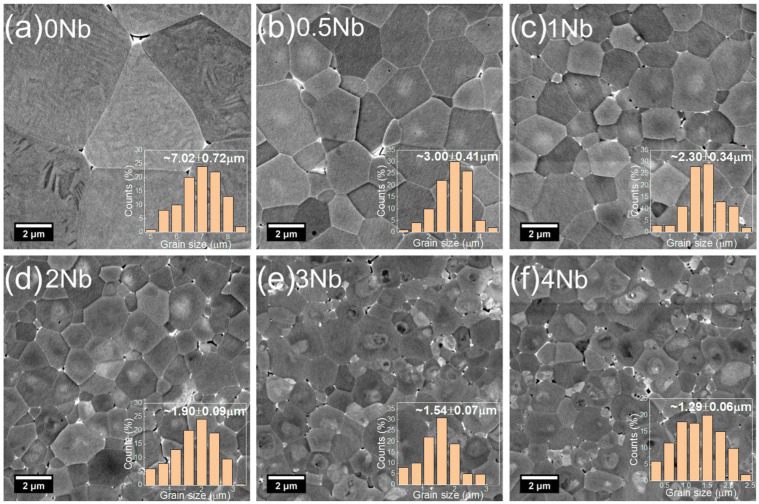
Scanning electron microscope images (back-scattered mode) and histograms of grain size distributions for 0.7BF–0.3BT–xNb ceramics with (**a**) x = 0, (**b**) x = 0.005, (**c**) x = 0.01, (**d**) x = 0.02, (**e**) x = 0.03 and (**f**) x = 0.04.

The influence of Nb-doping on the microstructure of the 0.7BF–0.3BT–xNb ceramics is illustrated by the SEM micrographs shown in Figure 2. Ordered ferroelectric domain patterns are clearly observed in the undoped 0.7BF–0.3BT ceramic, without any evident second phases, as shown in Figure 2a. The domain features diminish in size and regularity with Nb doping, even at the lowest level of 0.5 at%. These observations are consistent with the rhombohedral to pseudo-cubic transition induced by Nb substitution, as identified in the XRD results described above, which is associated with a gradual suppression of long-range ferroelectric ordering. Furthermore, there is a progressive reduction in grain size (from 7.0 to 1.3 µm), accompanied by the appearance of chemically heterogeneous core-shell type microstructural features in the Nb doped samples, which became more pronounced with the increase in Nb concentration as shown in Figure 2b–f. It is evident that the substitution of aliovalent Nb^5+^ ions influences the lattice diffusion and increases the activation energy for grain boundary migration [33]. Similar phenomena have been reported in previous studies of donor- or acceptor-doped BF–BT ceramics, in which the reduction in grain size may be induced by a solid solution impurity drag mechanism [34,35].

**Figure 3:**
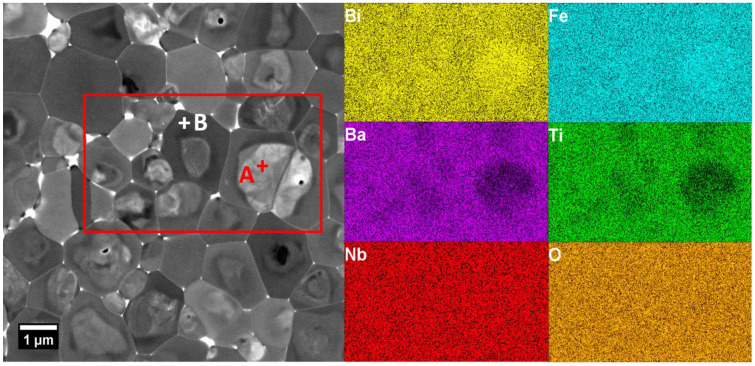
Back-scattered (BSE) SEM image and elemental mapping of the 0.7BF–0.3BT–4Nb ceramic. The red rectangular area defines the region used for micro-chemical mapping.

The occurrence of chemical heterogeneity is supported by EDS mapping of the elemental distribution in the 0.7BF–0.3BT–4Nb ceramic, as illustrated in Figure 3 and Table 2. These results indicate that Bi and Fe tend to segregate in the core regions, while Ba and Ti are enriched in the shell regions, i.e., a tendency for phase separation into BiFeO_3_- and BaTiO_3_-rich solid solutions. On the other hand, Nb and O appear to be distributed evenly within the characterized region. However, accurate determination of the distribution of Nb needs to be investigated further by other techniques due to the insensitivity of the EDS detector for this low dopant concentration. Bright features located at the triple points and along grain boundaries in the BSE images indicate the development of Bi-rich liquid phases during sintering, which may assist with densification. The existence of chemical heterogeneity in the form of core-shell type microstructural features has a significant impact on functional properties and also causes differences in crystal structure between the core and shell regions, as reported previously in BF–BT and other solid solutions such as 0.24BiFeO_3_–0.56K_0.5_Bi_0.5_TiO_3_–0.20PbTiO_3_ (BF–KBT–PT) [36]. Similar micro-chemical segregation effects were also evident for all of the Nb-doped BF–BT ceramics in the present study.

### 3.2. Dielectric and Ferroelectric Properties

**Figure 4:**
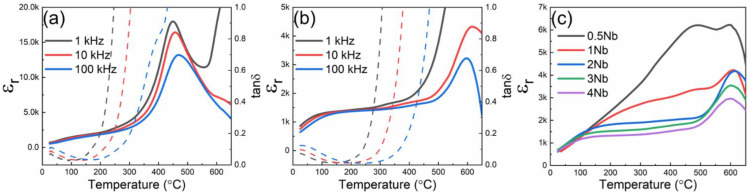
Temperature-dependence of relative dielectric permittivity (ɛ_r_) and loss (tan δ) for (**a**) 0.7BF–0.3BT–0Nb (**b**) 0.7BF–0.3BT–2Nb and (**c**) 0.7BF–0.3BT–xNb ceramics, measured at 10 kHz. Dashed lines in (**a**,**b**) indicate dielectric loss values.

The temperature dependence of dielectric permittivity (ɛ_r_) and loss (tan δ) of the 0.7BF–0.3BT–xNb ceramics are shown in Figure 4. The results obtained for the undoped 0.7BF–0.3BT ceramics exhibited a relatively sharp peak in permittivity at temperatures around 470 °C, Figure 4a, which is associated with the ferroelectric–paraelectric transformation at the Curie point. However, there was also a slight reduction in permittivity and increase of the peak temperature with increasing frequency, indicating a tendency for relaxor ferroelectric characteristics. The enhancement of dielectric loss at high temperatures and low frequencies is attributed to increasing contributions from electrical conductivity, as described by Hardtl [37]. In this respect, it is evident that the use of Nb-doping helped to shift the exponential rise in conductivity to higher temperatures, indicating the suppression of p-type electronic conductivity by the donor dopant. 

Assuming that the conduction mechanisms in BF–BT are similar to that of BiFeO_3_, we suppose that oxygen vacancies, caused by volatilization of bismuth oxide during sintering, may be reoxidized during cooling in air to form free electron holes, according to Equations (5) and (6), respectively.

(5) $$2{\mathrm{Bi}}_{\mathrm{Bi}}^{x}+3O_{O}^{x}\to 2V_{\mathrm{Bi}}^{///}+3V_{O}^{••}+{\mathrm{Bi}}_{2}O_{3}\left(g\right)$$

(6) $$2V_{O}^{••}+O_{2}\left(g\right)\to 2O_{O}^{x}+4h^{•}$$

Incorporating a donor dopant, such as Nb^5+^ substituted for Fe^3+^, provides a mechanism to suppress the formation of oxygen vacancies and therefore reoxidation cannot occur. In this case, the (relative) negative charge on a bismuth vacancy is compensated by the (relative) positive charge of the donor, rather than that of the oxygen vacancy, according to Equation (7).

(7) $$3{\mathrm{Bi}}_{2}O_{3}+3{\mathrm{Nb}}_{2}O_{5}\to 2{\mathrm{Bi}}_{\mathrm{Bi}}^{x}+4V_{\mathrm{Bi}}^{///}+6{\mathrm{Nb}}_{\mathrm{Fe}}^{••}+18O_{O}^{x}+2{\mathrm{Bi}}_{2}O_{3}\left(g\right)$$

According to the stoichiometry of this equation, two molecules of Bi_2_O_3_ may be volatilized without the formation of oxygen vacancies when three units of Nb_2_O_5_ are incorporated into solid solution, due to the necessary charge balance between the bismuth vacancies and the substituted Nb defects. Excessive Nb doping exceeds the solubility limit and leads to the formation of second phases, as noted in Section 3.1 above.

The main dielectric peak at 470 °C was strongly suppressed by Nb-doping, while a second peak appeared at elevated temperatures around 600 °C (Figure 4b). This second, higher temperature peak is attributed to the ferroelectric–paraelectric transformation in the BiFeO_3_-rich core regions, as identified in the microstructural observations reported above. A relatively stable dielectric plateau was formed between 150 and 500 °C for the Nb doped samples with x in the range of 2 to 4 at%, which is attributed mostly to the contribution from the shell regions. We suppose that incorporation of Nb leads to enhanced disorder of the local charge and lattice distortion, leading to nano-scale local random fields and the formation of polar nano regions (PNRs). The long-range ferroelectric order is suppressed, which induces relaxor-like behavior and the formation of the plateau region [38,39].

**Figure 5:**
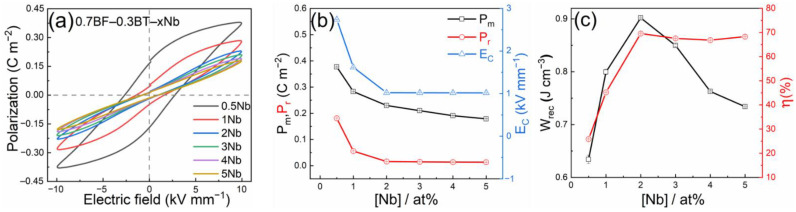
(**a**) Polarization–electric field (P–E) loops of 0.7BF–0.3BT–xNb ceramics, (**b**) maximum and remanent polarization (P_m_ and P_r_) and coercive electric field (E_C_) as a function of Nb concentration, (**c**) recoverable energy density (W_rec_) and efficiency (η) as a function of Nb concentration, measured at room temperature.

The polarization–electric field (P–E) loops of the BF–BT ceramics are shown in Figure 5a; the results for the undoped material are omitted from this plot due to excessive “rounding” effects caused by high leakage current and a tendency for electrical breakdown at high field levels. Both the maximum (P_m_) and remanent (P_r_) polarization values reduced dramatically with increasing Nb concentration in the range from 0.5 to 2 at%, followed by a gradual decrease in P_m_ and slight variations in P_r_ from 3 to 5 at%, as shown in Figure 5b. While P_r_ was already reduced to a negligible level at 2% Nb, further increases in Nb concentration resulted in continued reductions in P_m_, which led to progressive reductions in recoverable energy density, W_rec_. It is considered that the long-range ferroelectric ordering was disrupted, along with an evolution from nonergodic to ergodic relaxor ferroelectric behavior with increasing Nb concentration, yielding more reversible polarization switching characteristics. It was observed that donor doping with Nb led to improved breakdown strength, which is attributed to the combined effects of a reduction in the free carrier concentration and reduced grain size.

The compositional dependencies of the recoverable energy density (W_rec_) and efficiency (η) are shown in Figure 5c. The highest values of W_rec_ (0.9 J cm^−3^) and η (69.6%) at room temperature are achieved for the 0.7BF–0.3BT ceramics doped with 2 at% Nb; this material demonstrates the greatest potential out of those investigated in the present study and may be suitable for applications in energy storage capacitors and temperature-stable dielectrics.

**Figure 6:**
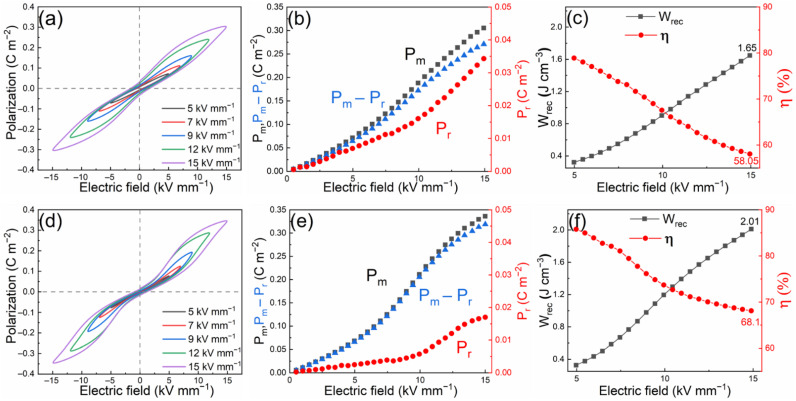
Polarization–electric field (P–E) loops of 0.7BF–0.3BT–2Nb ceramics under different electric fields at (**a**) 25 °C and (**d**) 75 °C. Maximum and remanent polarization (P_m_ and P_r_) and difference (P_m_ − P_r_) as a function of electric field for 0.7BF–0.3BT–2Nb at (**b**) 25 °C and (**e**) 75 °C. Recoverable energy density (W_rec_) and energy storage efficiency (η) as a function of electric field of 0.7BF–0.3BT–2Nb at (**c**) 25 °C and (**f**) 75 °C.

Figure 6a,d illustrate the P–E loops of 0.7BF–0.3BT–2Nb ceramics measured under various electric fields at 25 and 75 °C, respectively. At 25 °C, the nearly linear behavior of maximum polarization up to 15 kV/mm (P_m_) exhibits little sign of saturation, as shown in Figure 6b, whereas a gradual flattening with enhanced P_m_ at high fields (above 12 kV/mm) is observed in the measurements conducted at 75 °C, indicating a tendency for saturation. The remanent polarization is also reduced significantly at 75 °C, in comparison with that measured at 25 °C. These observations indicate a more reversible field-induced switching of the PNRs at elevated temperatures, leading to enhanced W_rec_ and η values, which improved from 1.65 J cm^−3^ to 2.01 J cm^−3^ and from 58% to 68% at 15 kV/mm, as the temperature increased from 25 to 75 °C. 

**Figure 7:**
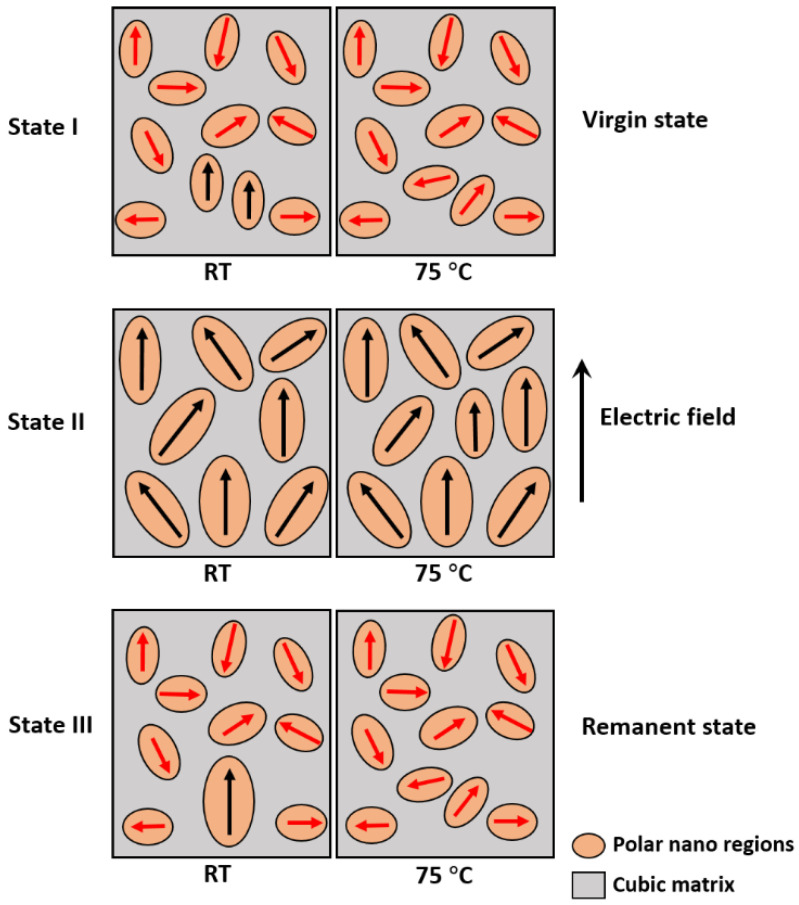
Schematic diagram illustrating the reorientation of PNRs under the influence of an applied electric field in the 0.7BF–0.3BT–2Nb ceramic, with both ergodic (reversible) and non-ergodic (partially irreversible) relaxor ferroelectric characteristics.

A schematic illustration of the changes in the nano-polar ferroelectric response for the 0.7BF–0.3BT–2Nb at different temperatures is shown in Figure 7. At room temperature, the random orientation of PNRs in the virgin state (State I) becomes ordered under an applied electric field (State II) and retains partial ferroelectric ordering in the remanent state (State III), i.e., the polarization switching is partially reversible and the material has a non-ergodic relaxor ferroelectric character [40]. Upon increasing the temperature to 75 °C, electric field-induced ferroelectric ordering of the PNRs also occurs, but the lower remanent polarization indicates that the random orientation is mostly recovered after the field is reduced to zero in State III, i.e., the polarization switching is more reversible and the material has an ergodic relaxor ferroelectric behavior [40]. 

## 4. Conclusions

The present paper demonstrates that undoped 0.7BF–0.3BT ceramics exhibit a rhombohedral phase and well-defined ferroelectric domain structures. Nb-doping induces an apparently pseudo-cubic structure, accompanied by core-shell microstructural features, even at the lowest substitution level of 0.5 at%. The presence of bismuth and niobium rich second phases at high Nb concentrations indicates a solubility limit of approximately 2 at%. Distinct peaks in the dielectric permittivity-temperature relationships for Nb-doped 0.7BF–0.3BT ceramics at temperatures of 470 and 600 °C are attributed to dominant contributions from the shell and core regions, respectively. The relatively high recoverable energy density (W_rec_ = 2.01 J cm^−3^) and the energy storage efficiency (η = 68%) were achieved in the 0.7BF–0.3BT binary system. These results demonstrate that Nb-doped BF–BT ceramics are promising lead-free dielectrics for next-generation pulsed power capacitor applications.

## Figures and Tables

**Table 1:** Lattice parameters of 0.7BF–0.3BT–xNb ceramics determined by full-pattern refinement. Numbers in parentheses indicate the uncertainty in the least significant digit.

	0 Nb	0.5 Nb	1 Nb	2 Nb	3 Nb	4 Nb	5 Nb
**Phase**	R3c	Pm$\bar{3}$m	Pm$\bar{3}$m	Pm$\bar{3}$m	Pm$\bar{3}$m	Pm$\bar{3}$m	Pm$\bar{3}$m
a = b = c (Å) *	4.0017(7)	3.9941(7)	3.9945(1)	3.9944(6)	3.9945(7)	3.9950(4)	3.9955(7)
**Cell volume (Å^3^)**	63.713(8)	63.720(4)	63.736(9)	63.734(5)	63.739(8)	63.762(2)	63.787(5)

* The lattice parameter for the 0 Nb composition corresponds to that of the rhombohedral phase [32].

**Table 2:** EDS point analysis (atomic %) of selected core (A) and shell (B) regions of the 0.7BF–0.3BT–4Nb ceramic shown in Figure 3.

	O	Bi	Fe	Ba	Ti	Nb
**Theoretical average**	60	14	13.44	6	6	0.56
**Point A (core)**	59.0	17.1	15.1	4.7	3.8	0.3
**Point B (shell)**	59.4	13.5	11.7	8.3	6.7	0.4

## Data Availability

Not applicable.
